# Enactive cinema paves way for understanding complex real-time social interaction in neuroimaging experiments

**DOI:** 10.3389/fnhum.2012.00298

**Published:** 2012-11-01

**Authors:** Pia Tikka, Aleksander Väljamäe, Aline W. de Borst, Roberto Pugliese, Niklas Ravaja, Mauri Kaipainen, Tapio Takala

**Affiliations:** ^1^NeuroCine, Department of Film, Television and Scenography, School of ARTS, Aalto UniversityHelsinki, Finland; ^2^Neuropsychology Laboratory, University of GrazGraz, Austria; ^3^NeuroGenetics Media Lab, CIANTPrague, Czech Republic; ^4^Department of Media Technology, School of Science, Aalto UniversityEspoo, Finland; ^5^Department of Social Research and Helsinki Institute for Information Technology, University of HelsinkiHelsinki, Finland; ^6^School of Business, Aalto UniversityHelsinki, Finland; ^7^School of Communication, Media and Information Technology, Södertörn UniversityHuddinge, Sweden

**Keywords:** enactive cinema, real-time fMRI, neurofeedback, social neuroscience, generative storytelling, implicit interaction, Brain Computer Interfaces

## Abstract

We outline general theoretical and practical implications of what we promote as *enactive cinema* for the neuroscientific study of online socio-emotional interaction. In a real-time functional magnetic resonance imaging (rt-fMRI) setting, participants are immersed in cinematic experiences that simulate social situations. While viewing, their physiological reactions—including brain responses—are tracked, representing implicit and unconscious experiences of the on-going social situations. These reactions, in turn, are analyzed in real-time and fed back to modify the cinematic sequences they are viewing while being scanned. Due to the engaging cinematic content, the proposed setting focuses on *living-by* in terms of shared psycho-physiological epiphenomena of experience rather than active coping in terms of goal-oriented motor actions. It constitutes a means to parametrically modify stimuli that depict social situations and their broader environmental contexts. As an alternative to studying the variation of brain responses as a function of *a priori* fixed stimuli, this method can be applied to *survey the range of stimuli* that evoke similar responses across participants at particular brain regions of interest.

## Introduction

“David observes Sally entering a coffee room filled with lively conversation of colleagues. Immediately the conversation ends and everybody leaves hastily. Sally is left alone. David sees how she fights the tears while sipping her coffee, and cannot help feeling sorry for her. The scene repeats every day. Then once again, the coffee room is crowded. David is already expecting Sally's entrance. Suddenly someone looks directly at David, as if realizing his presence. This time, as Sally enters and everybody else leaves, the person who has shared a look with David hesitates. Glances at David but then leaves together with others, which makes David feel disappointed. Next time during the coffee break, the person with whom David earlier shared a look, unexpectedly remains and greets Sally. The gratefulness on Sally's face, and a quick glance that the fellow worker shares with David, makes David feel that his engagement with the events has made a difference.”

Consider that David, instead of being physically in the same space as Sally *and* despite of lying motionless in an fMRI scanner, is nevertheless *enactively* engaged in the setting. That is, he not only reacts to but also influences the emotional behavior of the screen characters. According to our vision, this setting addresses *socially synchronized and shared everyday experiences* online by means of engagement characteristic for cinema viewing.

### Neuroscience of social interaction

In experimental settings social behavior is typically stripped out of its situational and embodied contexts due to methodological reasons, thus risking their ecological validity (Neisser, 1976; Gibson, 1979). Recent paradigms of social neuroscience experiments try to overcome these problems by, for example, letting participants in a functional magnetic resonance imaging (fMRI) scanner interact with someone inside another scanner (Montague et al., 2002), or outside of the scanner (Redcay et al., 2010). Also magnetoencephalographic (MEG-to-MEG) real-time interaction recordings have been successfully conducted (Baess et al., 2012). Further, the development of two-person fMRI head-coils allows a major step forward in introducing physical presence and intimacy to online interaction settings in fMRI (Lee et al., 2010, 2011). But adding another person into the scanner still leaves these settings far from capturing context-dependency of social behavior. Since the biological brain continuously simulates the phenomena of surrounding world (Rizzolatti and Arbib, 1998; Iacoboni et al., 2005; Rizzolatti and Fabbri-Destro, 2008; Cattaneo and Rizzolatti, 2009; Hari and Kujala, 2009), brain activity cannot be fully understood without a model that addresses even the dynamics of the social environment.

### Neurocinematics

Cinema serves the interest of social neuroscience because it simulates life by its very nature (Tan, 1996; Mar and Oatley, 2008; Tikka, 2008; Grodal, 2009). *Neurocinematics*, so termed by Hasson et al. (2008b) refers to neuroimaging experiments that use cinematic stimuli to study human behavior. So far, neurocinematic experiments have shown that different viewers' attention to socially determining aspects of the story, often communicated by facial expressions and bodily gestures, is highly correlated (Bartels and Zeki, 2003, 2004; Hasson et al., 2004, 2008a,b, 2010; Bartels et al., 2008; Jääskeläinen et al., 2008; Lahnakoski et al., 2012).

Regarding cinema-viewing in fMRI, we consider two particular conditions essential to social validity. The method of *unconstrained free viewing* in fMRI allows viewer's emotional immersion into the cinematic world without disturbing breaks. This condition is not different from the reported experiences of presence in virtual reality experiments (Hoffman et al., 2003; Sanchez-Vives and Slater, 2005; Baumgartner et al., 2008). The viewer's engagement with the fictional characters may be understood as parasocial interaction in the sense of Horton and Wohl (1956), or as a kind of inter-reality, an experience that converges real and fictional worlds (Riva et al., 2010). As another aspect of social validity we take the characteristic of immediate two-way responsiveness. This is difficult to achieve with traditional cinema material with a fixed storyline. While the paradigm of interactive cinema has introduced various means of combinatory and generative narrative solutions to this issue, it has another problem: the conscious decision-making expected from the viewers often interrupts the narrative flow. The dilemma we suggest to solve with the enactive cinema setting is how to model participants' engagement in the social situations created by cinematic scenes without destroying the emotional immersion.

### Enactive cinema

While regular cinema may be regarded as simulation of life situations, enactive cinema takes the simulation one step further by letting the viewer's experience influence the narrative in real-time. Being *enactive* refers to engagement that is more holistic than being interactive. The origin of the notion goes back to the idea of embodied interaction between a subject and another entity as discussed by Varela et al. (1991). In an enactive system, changes in the psychophysiological reactions of participants (enactors) that are assumed to represent implicit and unconscious reactions of the mind, determine the changes made to the narrative presentation in real-time (Kaipainen et al., 2011). The related concept of “enactive cinema” was first introduced in the context of cinema installation “Obsession” (Tikka, 2005). This installation relied on (1) the tracking of spectator's real-time physiological responses, such as heart rate and electrodermal activity, which controlled (2) the algorithm-driven montage of the cinematic expression, which again influenced the spectator's experience, and so on (Tikka et al., 2006). The continuous follow-up of the changes in the participants' bodily responses drives algorithmic modification of the cinematic narration in such a manner that the viewpoints of the ongoing events may change, for example, from suspense to romantic expectations. As a distinction to what is typically called interactive media, in enactive media the participants are not directly in control of the narrative. Enactive cinema can be regarded as modeling socio-emotional dialogs by means of modifying situational factors as a function of participants' experience. The advantage of enactive cinema over conventional linear narration for real-time social neuroscience experiments lies in the systemic interdependence due to two-way feedback, which addresses the socio-emotionally engaging nature of cinema as a simulation of the situatedness in the “real” world.

### Real-time physiological feedback

Part of the methodology that can be used to implement an enactive cinema system stems from the vast body of experimental and clinical research using physiology-based real-time feedback since the 1970's (e.g., Nowlis and Kamiya, 1970; Manuck et al., 1975). By presenting participants with feedback of their own physiological state (heart rate, breathing, brain activity, etc.) control over these states can be learnt (e.g., deCharms et al., 2004; Weiskopf et al., 2004). Recently, real-time functional magnetic resonance imaging (rt-fMRI; Cox et al., 1995) has allowed neural activity of multiple brain regions to be used as feedback (see Weiskopf, 2011, for overview). Due to the development of real-time connectivity analysis (Lee et al., 2012) and multivariate classification (Hollmann et al., 2011) real-time fMRI can go beyond mere manipulation of region-of-interest based activity by allowing network analyses and actual prediction of behavior. These neurofeedback methods seem particularly suitable for the investigation of emotions and social behavior (Posse et al., 2003; Johnston et al., 2010; Sarkheil et al., 2011; Veit et al., 2011).

The measures of neural activity can be complemented by peripheral physiological and behavioral signals that give access to emotional and attentional states: e.g., heart-rate and electrodermal activity may indicate emotional arousal, facial electromyography (EMG) activity can index affect, and eye-tracking inform about the direction of overt attention (see e.g., Davidson, 2004; Ravaja, 2004; Heller et al., 2011). Real-time feedback has been explored in various virtual reality settings. For example, eye-tracking has been used to reflect participants' real-time response to the behavior of a virtual character in fMRI (Wilms et al., 2010). Outside of scanner, the motion-capture experiment of Pugliese and Lehtonen (2011) explored a feedback loop of action and reaction between a human participant and a virtual character by means of imitation and designed rules. In the video game *Ghost in the Cave* the player can control the emotional state of a virtual character (avatar) by using nonverbal acoustical or motion cues (Rinman et al., 2004). Moreover, emotionally adaptive games (e.g., *Emoshooter*) dynamically change their design depending on the player's emotional state (Kuikkaniemi et al., 2010).

However, the proposed enactive approach goes beyond the previously described paradigms by combining physiological feedback techniques with socially engaging cinematic stimuli. While sharing many techniques with the rapidly developing area of Brain Computer Interfaces (BCI) for neuroscience of social interaction (Mattout, 2012) as well as with unconscious man-machine control loops (e.g., Kaplan et al., 2005), the enactive cinema emphasizes more holistic human-system dialogs.

## Enactive cinema for social real-time neuroscience

Rather than studying the variation of brain responses as a function of a-priori fixed stimuli we suggest the study of brain activity as a function of dynamically adapted stimuli. Rt-fMRI feedback reflecting the viewer's idiosyncratic brain activity patterns, potentially combined with their peripheral physiological responses, can be applied to modify cinematic stimuli that address ranges of socially engaging situations. The comparison of parametric variations in cinematic stimuli with the synchronous brain data will augment the understanding of the dynamical context-dependency of the social mind.

### Enactive cinema system

**Figure 1:**
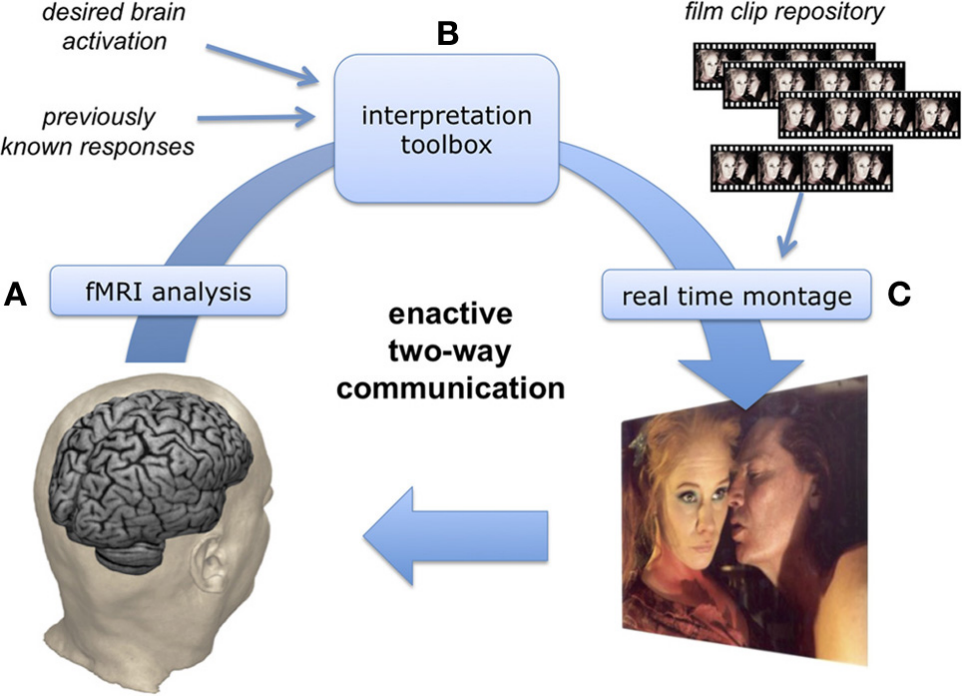
**Diagram of the enactive setting: (A) the viewer's physiological responses are analyzed in real-time, (B) an “interpretation toolbox” compares them with previously known behaviors and selects cinematic elements that are expected to lead toward a desired response, (C) real-time montage produces continuous cinematic stimulus projected back to the participant, whose brain responses are scanned (A), and so on**.

The enactive cinema system includes (1) real-time acquisition and (2) analysis of physiological response data, which is further fed into an (3) “interpretation toolbox” that modifies the stimulus stream so as to trigger desired responses based on previous knowledge of brain behavior (see Figure 1).

### Enactive rt-fMRI feedback

The availability of real-time analysis methods is constantly increasing, thereby greatly enhancing the possibilities of an enactive cinema system. Next to the established real-time analyses of ROIs, several other properties of fMRI data can be exploited, such as functional connectivity (e.g., rt-ICA; Ma et al., 2012), effective connectivity (Lee et al., 2012), pattern classification (Hollmann et al., 2011; LaConte, 2011), as well as a combination of brain data and peripheral physiology (Wilms et al., 2010; Voyvodic et al., 2011). This entails that feedback to the system can be more than a one dimensional increase-decrease relationship of a single region, allowing more fine-grained reflection of the mental state. For example, using real-time ICA the involvement of specific functional networks can be defined. While a single region can be involved in several different cognitive functions, the identification of a functional network will give the system more information about the ongoing cognitive processes, allowing for more detailed feedback. The accuracy of the identification of the cognitive, emotional, and motivational states of an individual can be enhanced by combining several physiological indices with the rt-fMRI data, as the advantages of response patterns (or profiles) over single physiological measures are well established (e.g., Ravaja, 2004). For example, employing several physiological signals, neural networks (e.g., probabilistic neural network) have successfully been trained to recognize emotions when viewing films (Arroyo-Palacios and Romano, 2010). As an example of potential settings, assuming that each participant's brain activation correlates to some extent with a particular type of experience (e.g., fear; Ehrsson et al., 2007), one may optimize the similarity across different participant's brain activations by means of stimuli personalized in real time.

### Enactive cinematic stimuli

We envision two technical solutions for the real-time presentation of enactive cinematic content in fMRI: (1) the fine-grained recombination of existing cinematic elements, and (2) algorithmic generation of cinematographic characters and images by adapting methods from the fields of animation, gaming and virtual reality research (e.g., Parsons and Courtney, 2011).

The recombination of cinematic elements in real-time allow varying the contextualizing conditions of social interaction within the experimental focus. Its prerequisite is the rich time-coded metadata associated with the content, which enables the retrieval of video footage from a content database and the sorting of this footage into a coherent sequence. The rough annotation of the content with attributes (or “tags”) such as low level perceptual features (e.g., visual, auditory), ecologically valid features (e.g., hands, faces, basic emotions) and higher level features (e.g., social, psychological, cultural) may not suffice to the generation of fine-grained coherent narrative sequences that meet the criteria of social validity.

Based on the ontospace approach (Kaipainen et al., 2008; Kaipainen and Hautamäki, 2011), a metadata approach to narrative has been elaborated (Pugliese et al., 2012), where meaning-bearing annotations of content are described by means of graded values, referring to the prominence of particular content-related features. These values may correspond to above mentioned annotations or, for example, rules of cinematic storytelling (e.g., flashbacks, emotion cues, or temporal ellipses). They are conceived of as *narrative dimensions* that altogether define a space of narrative metadata, allowing each narrative element to be described in terms of its narrative coordinates (Pugliese et al., 2012). The advantage of this approach is that it allows a dynamical stimulus montage as an element-to-element trajectory through the narrative medatata space, which in turn is shaped by a parametrically controllable *perspective*, a set of weights associated with one for each dimension. In this space elements similar to each other with respect to the weighted dimensions lie in each other's proximity. Insofar the metadata describes the socially valid aspects of the narration, the approach guarantees a stimulus montage that is not only continuous but even socially coherent. Enactive cinema installation *Obsession* (Tikka, 2005) can be regarded as a preliminary proof of concept.

The described method can also be applied to generate desired behavior of *dynamically controlled stimulus characters*, or avatars that can be used to model a partner in a social dialogue. In this case, content-describing narrative dimensions correspond to generative parameters of the avatar. This approach relies on findings of recent fMRI experiments that have revealed the sensitivity of brain functions associated with viewing artificial human-like agents in comparison to humans (Moser et al., 2007; Chaminade et al., 2010; Cheetham et al., 2011; Saygin et al., 2011), confirming the observation of the “uncanny valley” by Mori (1970). However, as the recent technological developments render virtual avatars more human-like than ever before (Rizzo et al., 2004; Alexander et al., 2010), we envision that the technology can eventually bridge the uncanny valley.

### Toward implementation of enactive cinema in fMRI

How enactive cinema may be harnessed to study complex social settings in fMRI can be exemplified on the basis of Singer et al. (2006), who showed that after perceiving two people being fair or unfair in an economical game and then seeing both persons receiving painful electrical shocks, two types of response patterns emerged in the participants. The female subjects showed empathy-related brain activity to seeing both fair and unfair persons receiving pain, while the males showed reward-related activity to seeing an unfair person receiving pain (revenge). Applying the enactive cinema setting to this phenomenon, it is possible to study the context-dependency of empathy and revenge. Based on the real-time emerging response patterns in e.g. anterior insula (empathy) or orbito-frontal cortex (revenge) the stimuli can be adapted online for each individual. For example, when participants show a low blood oxygen level dependent (BOLD) signal in anterior insula the system computes, using algorithms and metadata (prior knowledge), dynamical modifications of the cinematic stimulus (e.g., adding personal background information on the unfair person) in order to increase activity/empathy. By incrementally adapting the stimulus, the desired response can be obtained in both males and females. Empathy can then be studied by looking at similarities and differences between the stimuli (e.g., additional information, emotion cues, context, etc.) that made brain responses of the males similar to those of the females.

Similarly, in the case described in the introduction, the fMRI participant's physiological epiphenomena would be associated with David's empathy toward Sally when seeing her socio-emotionally painful situation. Changes in David's biometric measures of arousal as well as neural activity of his limbic regions, perhaps even in some of the brain networks that relate to moral judgments (Parkinson et al., 2011), could be interpreted as indications of changes in his attitude toward the depicted scene. Depending on the goals of the experiment, the stimulus stream could be modified by priming David's perspective to Sally for example by adding explanatory background information concerning Sally's previous activities, or by manipulating the degree of mental violence of the scene, or adjusting the duration of the painful situation.

Due to the two-directionality of the enactive system, the online adjustment of the cinematic stimuli described above on the one hand, and the real-time manipulation of neural activations on the other hand, allow a novel kind of analysis of the brain's functional activity and connectivity versus parametric changes in the stimulus stream.

## Conclusion

We have proposed an approach to neuroscience of social interaction that focuses on shared social engagement in dynamical contexts. It is based on the potential of cinema to model everyday situations of life. In order to apply cinematic stimuli in fMRI settings we have suggested the method of generation of cinematic stimulus stream in real-time by means of re-combining existing elements algorithmically, with potential supplementary elements such as animations or avatars. The stream is controlled by the participants' enactivity, i.e., their psychophysiological epiphenomena of experience. For the neuroscience of social interactions enactive setup offers for the first time a means to generate contextualized, socially valid stimulus material under dynamical control, allowing observation of brain activity in the course of a social dialog. Although we acknowledge many technological challenges, however, due to the rapidly evolving real-time implementations both in the field of neuroimaging as well as that of computer generated audiovisual media, we envision that the implementation of an enactive system is only a matter of time.

### Conflict of interest statement

The authors declare that the research was conducted in the absence of any commercial or financial relationships that could be construed as a potential conflict of interest.
